# Effects of Intrinsic and Orbital Angular Momentum on the Swimming Individual and Relay Starts Performance

**DOI:** 10.5114/jhk/202263

**Published:** 2025-09-23

**Authors:** Enrique Navarro, Alfonso Trinidad, Santiago Veiga

**Affiliations:** 1 Health and Human Performance Department, Universidad Politécnica de Madrid, Madrid, Spain.; 2 AquaLab Research Group, Education and Educational Innovation Department, Universidad Europea de Madrid, Villaviciosa de Odón, Madrid, Spain.; 3 Sports Department, Universidad Politécnica de Madrid, Madrid, Spain.

**Keywords:** kinematics, biomechanics, competition, track start, body rotation

## Abstract

There is currently a lack of knowledge about the rotational component of competitive starting techniques using starting blocks equipped with an adjustable back plate and its effect on water entry. The aim of the present study was to examine the angular momentum components of the current competitive swimming starts and to compare the contribution of the body segments to the rotational component of the individual kick start and the relay step start techniques. The block and aerial starting movements of eleven competitive swimmers during an individual and relay start from an Omega OSB11 were filmed at 120 Hz. The total body and the segmental contributions to the intrinsic and orbital components of the angular momentum were computed. Regardless of the type of the start, the orbital rotation of the body segments around the swimmer's centre of mass accounted for a large proportion (between 89 and 91%) of the total angular momentum. At the take-off, the total angular momentum was greater in relay step starts than in individual kick starts (η2 = 0.71). However, the competitive swimmers showed larger lower limb entry angles during the individual kick start (291.5 ± 1.8° vs. 282.2 ± 3.4°), related to a greater segmental contribution of lower limbs (56.5% ± 2.8) to the total angular momentum (η2 = 0.76). The adjustable back plate of the block provided a greater rotational component for the lower limbs in the individual kick start (compared to the relay step start), which assisted swimmers in achieving a better body posture at the water entry.

## Introduction

Swimming starts are one of the key components of swimming races not only due to their quantitative contribution to the race time (more important in sprint events) (Born et al., 2021), but mainly due to the greater swimmers’ forward velocities when diving-off the starting block than during clean or surface swimming (Veiga and Roig, 2017). This is the reason why the start techniques have attracted the interest of researchers over the years looking for optimizing the starting movements for the swimmers’ overall performance.

The start techniques differ depending on the type (individual or team relay) of swimming races. In the individual races, the use of the starting blocks with an adjustable back plate makes the kick start technique (Honda et al., 2010) the most common in these events. In this technique, according to World Aquatics rules, swimmers adopt a stationary track position (rear lower limb supporting the back plate and the front lower limb in the front part of the starting platform) before the starting signal. In relay races, on the other hand, swimmers can perform preparatory movements on the starting block provided the feet do not lose touch with the starting platform before the preceding team-mate touches the wall. This is the reason why most swimmers in relay races adopt the so-called step techniques (Qiu et al., 2021a) where they perform one or two steps from the rear to the front part of the starting platform before diving-off. Regardless of the start type, swimmers must aim for maximizing the horizontal impulse (Takeda et al., 2010) and, therefore, achieving great horizontal velocities at the take-off (García-Ramos et al., 2015). This will allow them to maximize the flight time and, consequently, the horizontal distance travelled by their centre of mass before the water entry.

However, at the same time of maximizing the horizontal displacement during the aerial start phase, swimmers should obtain a rotational force application during the block phase that would modify body orientation during the flight (Mclean et al., 2000). This angular momentum around the center of gravity remains conserved during the flight phase and would allow swimmers to minimize the hydrodynamic drag forces at the water entry (Houel et al., 2013). In fact, previous evidence suggests that greater values of angular momentum at the take-off increase the body rotation during the flight and provide swimmers with a steeper water entry (McLean et al., 2000; Vantorre et al., 2010). In the individual kick start, forces exerted by the rear lower limb on the back plate should mainly provide this rotation component (Taladriz et al., 2016), whereas in the relay step techniques, this may be achieved by the swimmers’ preparatory movements before the take-off (McLean et al., 2000).

When calculating the angular momentum of the human body, both the intrinsic or local rotation of each body segment around its centre of mass and the orbital or remote rotation of the segment around the centre of mass of the body should be taken into account (Bahamonde, 2000; Landau and Lifshitz, 1974). In fact, in many sport techniques, the rotational component of a given body segment could rely more on its linear momentum over the body centre of mass than segment rotation itself (see Annex 1). This is the reason why previous studies have examined both intrinsic and orbital angular components when examining rotational sport techniques (Bahamonde, 2000; Miller et al., 1985), although none of them in swimming starts. Mclean et al. (2000) examined the role of the step techniques on the angular momentum of relay starts, but, at that time, no starting blocks with an adjustable back plate and a greater platform surface were used. Other studies examined the differences in angular momentum between different individual start techniques. However, some of the employed start techniques such as the grab start (Taladriz et al., 2016) or the flat start (Vantorre et al., 2010) are not currently employed by competitive swimmers. At the moment, a gap in knowledge exists about the rotational component of the competitive start techniques and its consequences on the water entry. Therefore, the aim of the present study was to examine the angular momentum components of the current competitive swimming starts and to compare the contribution of particular body segments to the rotational component of the individual kick start and the relay step start techniques.

## Methods

### Participants

Data collection was done in a 50 x 25-m indoor swimming pool with water temperature at 27°C, located in the Madrid High Performance Centre (Spain). Experimental procedures were approved by the Institutional Review Board of the Universidad Politécnica de Madrid, Madrid, Spain (approval code: 2020-080; approval date: 19 November 2020) and respected the principles of the Declaration of Helsinki (WMA, Oct. 2013). Eleven swimmers (5 males and 6 females) belonging to the National Swimming Federation training programs volunteered to participate in the study. Swimmers had at least five years of training experience in a program averaging nine in-water sessions (≈16–18 h), three dry-land sessions (≈3–5 h) per week and their standardised performance level belonged to level two and level three ties (Ruiz-Navarro et al., 2023). In particular, the range of best times in their respective best events (50-m pool) were from 2:06.83 (200-m individual medley men) to 16:33.44 (1500-m freestyle women). Their age was 17.10 ± 1.26 and 16.12 ± 1.56 years (age range: 14.9 to 18.4 years), body height 175.38 ± 2.57 and 174.82 ± 6.43 cm, and body mass 63.38 ± 6.66 and 66.60 ± 5.75 kg for the male and female group, respectively. Prior to the experiment, a written informed consent form was signed by the swimmers, and for those aged under 18, the consent form was signed by their parents or legal guardians.

### Design and Procedures

#### Data Collection

After a standardised warm up of approximately 10 min of dry-land exercises and 20 min of in-water swimming, and during the usual training schedule, swimmers performed 6 x 25-m swimming trials at maximum effort with three-minute rest intervals in between. Trials began from an official Omega OSB11 (Swiss Timing Ltd., Switzerland) starting to mimic the competition conditions and swimmers employed the individual kick start or the relay step start technique, respectively, in each half of trials. The order of the three repetition blocks was randomized and the best swimming start (measured at 15 m) for each condition (individual or relay start) was selected for further analysis. As indicated in Figure 1, the individual kick start was characterised by swimmers adopting a stationary position before the starting signal with at least one foot at the front of the starting platforms. In the relay step start, swimmers performed a lower limb step from the rear to the front part of the starting platform at the same time of a circular backswing with upper limbs before diving off the wall. These preparatory block movements of the relay start could be freely done provided at least one foot was in contact with the starting platform before the preceding team-mate touched the wall.

**Figure 1:**
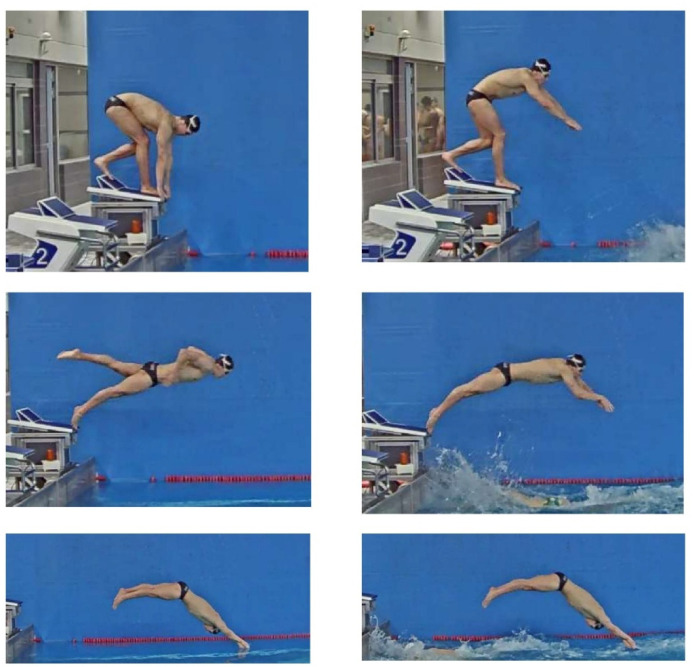
Key events (initial position, take-off and entry) for the individual (left) and relay (right) start techniques.

Trials were filmed from a lateral view following the set-up of Qiu et al. (2021a, 2024). A video camera (Casio, EX ZR1200 BK, Japan) sampling at 120 Hz and mounted on a tripod at 2 m from the starting wall and 3.5 m away from the centre of the swimming lane was used for the starts filming. The field of the view was calibrated using a quadrangular structure of 2 m high x 2 m wide located in the middle of the swimming lane, above the water surface, and containing eight control points. Direct Linear Transformation algorithms (Abdel-Aziz et al., 2015) were employed to convert the screen coordinates into real space two dimensional coordinates. The origin of the reference system was located in the starting wall at the water surface, with height and distance of the starting direction representing positive axes.

#### Data Processing

The present study analysed the swimmer’s movements on the block and aerial start phases. The starting movements were defined from the first observable movement on the block to the instant the swimmer’s hands contacted the water surface after the flight. The duration of each start trials was normalized to 0–100% by linear interpolation. Twenty-one body landmarks (vertex, cervicale, the hip segmentation planes (HSP) intersection, right and left acromion, right and left radiale, right and left stylion, right and left 3^rd^ dactylion, right and left iliospinale, right and left tibiale, right and left sphyrion, right and left heel, and right and left toe tip) were manually digitized in each of the video frames to define fourteen segments according to the Zatsiorsky-Seluyanov model, modified by De Leva (1996). Real-space digitized coordinates for each body landmark were digitally filtered using a low-pass fourth-order Butterworth filter, with the frequency showing the least autocorrelated residuals for each body landmark selected as a cut-off frequency (between 6 and 9 Hz). The accuracy of the digitizing procedure by the same observer and the Root Mean Squared Error estimation of the coordinates reconstruction using the same camera set-up was reported in previous research (Qiu et al., 2021a).

#### Definition of Variables

**Figure 2:**
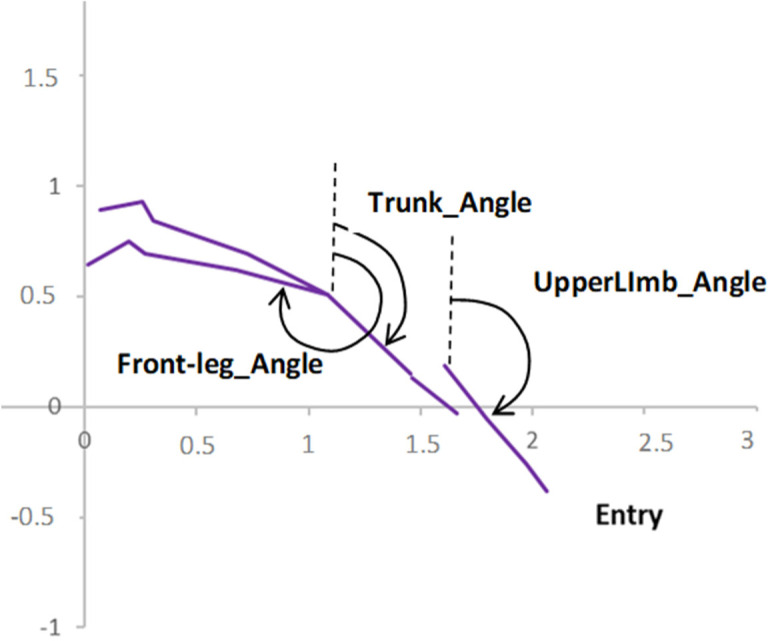
Definition of the attitude of body segments in relation with the vertical.

The swimmers’ body segment angles at the take-off and entry instants were calculated as the clockwise angle between the segment and the vertical in degrees (Figure 2). Each segment was regarded as a vector from the most proximal to the most distal joint. The trunk segment was defined as a vector from the HSP intersection to the cervicale. Lower limb segments were defined as the vector from the right or the left iliospinale to the right or the left sphyrion, respectively. Upper limb segments were defined as a vector from the right or the left acromion to the right or the left stylion.

The angular momentum of the body segments was calculated as the summation of the intrinsic or local and the orbital or remote angular momentum (Equation 1) (Landau and Lifshitz, 1974). While the intrinsic momentum is due to the rotation of the segment around its own centre of mass (CMs), the orbital momentum is (Equation 2) produced for the movement of the CM of the segment around the centre of mass of the body (Equation 3).


$$L_{CMb}^{s}=L_{CMs}^{i}+L_{CMb}^{o}\;\left(Equation\;1\right)$$

$$L_{CMs}^{i}=I_{x}\omega_{x}\vec{i}+I_{y}\omega_{y}\vec{j}+I_{z}\omega_{z}\vec{k}\;\left(Equation\text{ 2}\right)$$

$L_{CMs}^{i}$ : *intrinsic AM about the segment around its centre of mass I_x_; I_y_; I_z_ : moments of inertia around the principal axis of the segment ω_x_;ω_y_;ω_z_: components of the angular velocity around the principal axis of inertia*

$\vec{i};\vec{j};\vec{k}$: *unit vectors of the principal axis of inertia*>


$$L_{CMb}^{o}={\vec{r}}_{CMs}\times m_{s}{\vec{v}}_{CMs}\;\left(\text{Equation 3}\right)$$

$L_{CMb}^{o}$: *orbital AM around the body centre of mass*

${\vec{r}}_{CMs}$: *position vector of the centre of mass of the segment about the CMb*

×: *vectorial product*

$m_{s}{\vec{v}}_{CMs}$: *linear momentum of the segment related to the CMb*

The angular momentum of each segment was normalized by its respective length and mass (Taladriz et al., 2016) following equation 4. The normalized angular momentum of the body was the summation of the normalized angular momentum of the segments. In addition, the ratio of each body segment to the total body angular momentum as well as the ratio of the intrinsic component to the total body angular momentum were calculated. For computation, the ratio of the lower limbs was the sum of the thigh, shank and foot segments, whereas the ratio of the upper limbs was the sum of the arm, forearm and hand segments. A spreadsheet was developed for all the angular momentum computation.


$$NL_{CMb}^{s}=\left(\frac{L_{CMb}^{s}}{M\times H^{2}}\right)10^{3}\left[s^{-1}10^{-3}\right]\;\left(\text{Equation 4}\right)$$

$NL_{CMb}^{s}$: *normalized angular momentum of the segment*

$L_{CMb}^{s}$: *angular momentum of the segment*

*M: mass of the subject*

*H: height of the subject*

### Statistical Analysis

Descriptive statistics were performed for calculating the mean and mean standard error (SE) of 1) the body segment angles and 2) the intrinsic and orbital components of the respective body segments and the total body angular momentum. A multivariate analysis of variance (MANOVA) was employed to analyse the effect of the type of the start (individual kick start or relay step start) and the start phase (take-off and entry) on each dependent variable. After studying the main effects, a pairwise comparison of the variables was carried out, correcting the Type II error using the Bonferroni adjustment. The Levene's test was previously applied to check the homogeneity of variance. The effect size was measured using partial eta squared (η^2^) and it was interpreted according to the thresholds proposed by Olejnik and Algina (2003) (0.01 = small, 0.06 = medium, and 0.14 = large). All statistical analyses were conducted with SPSS 15.0 (SPSS Inc., Chicago, IL, USA) with the significance level set at 0.05.

## Results

Fifteen-metre times for the best individual and relay start of each swimmer during data collection were 7.14 ± 0.52 s and 6.67 ± 0.50 s, respectively. The body segments’ orientation during individual and relay start techniques revealed a significant effect for the front (F_2,8_ = 16.71, *p* = 0.001, η^2^ = 0.81) and rear (F_2,8_ = 13.66, *p* = 0.003, η^2^ = 0.77) lower limbs, for the upper limbs (F_2,8_ = 6.20, *p* = 0.024, η^2^ = 0.61), but not for the trunk-head (F_2,8_ = 2.94, *p* = 0.110, η^2^ = 0.42) at the take-off and entry instants (Table 1). These represented greater angles at the water entry for both the front and rear lower limbs in the individual than in relay starts, in addition to a greater rotation of the front lower limb from the take-off to the entry instants in the individual kick start.

**Figure 3:**
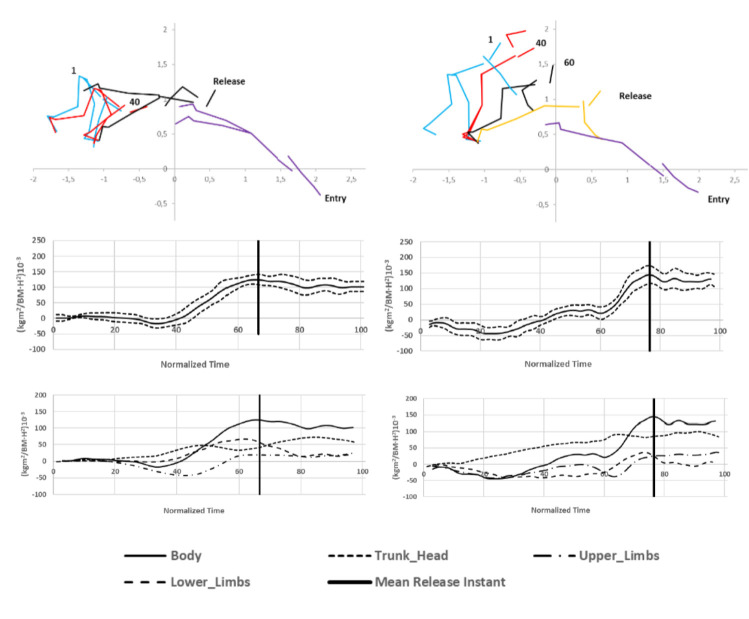
Total body angular momentum (above, mean ± standard error) and segments’ contribution (below) during individual (left) and relay (right) swimming start techniques.

The total angular momentum of relay starts at the take-off was greater than in individual kick starts (F_2,8_ = 12.71, *p* = 0.003, η^2^ = 0.71) (Table 2). Regardless of the start type, angular momentum reached low negative values (counterclockwise rotations of the segments) during the first 40% of start time and then turned into positive values (Figure 3), with maximum angular momentum of 152.9 ± 4.4 x 10^−3^∙s^−1^ for the relay step start and 127.8 ± 6.5 x 10^−3^∙s^−1^ for the individual kick start around the take-off.

**Table 1:** Angles (in degrees) of body segments to the vertical during the individual and relay swimming starts (mean ± SE).

		Individual		Relay	*p* value	Effect size
Trunk	Take-off	95.6 ± 2.1		92.7 ± 2.1	0.105	0.265
	Entry	129.9 ± 1.3		131.7 ± 2.1	0.271	0.133
	Increase	34.3 ± 2.4	<	39.0 ± 3.2	0.031	0.419
Front lower limb	Take-off	238.6 ± 1.5	<	247.3 ± 2.8	0.001	1.000
	Entry	291.5 ± 1.8	>	282.2 ± 3.4	0.001	1.000
	Increase	52.8 ± 2.6	>	34.9 ± 4.8	0.001	0.945
Rear lower limb	Take-off	270.6 ± 2.3	>	250.1 ± 3.3	0.001	0.738
	Entry	294.3 ± 1.7	>	282.4 ± 3.3	0.001	0.697
	Increase	23.7 ± 3.4	<	32.3 ± 4.3	0.037	0.399
Upper limbs	Take-off	174 ± 19.6	>	133.4 ± 8.3	0.019	0.473
	Entry	133.2 ± 2.4		134.6 ± 2.5	0.379	0.087
	Increase	−40.8 ± 20.2	<	1.2 ± 8.9	0.013	0.512

Note: < and > denote statistical increments of body segment angles from the take-off to entry positions (p < 0.05)

The contribution of body segments to the total angular momentum (Table 2) indicated that swimmers performing the relay start generated most of the angular momentum with the trunk-head at the take-off, followed by the lower and the upper limbs. However, in the individual kick start, the main contribution to the angular momentum at the take-off was from the lower limbs, while the trunk-head and upper limbs reached smaller contribution (Figure 3, Table 2). The contribution of the trunk-head to the total angular momentum was greater in the relay than in the individual starts (51% ± 3.6 vs. 28% ± 3.7, F_2,7_ = 53.53, *p* = 0.000, η^2^ = 0.94), but the one generated by lower limbs was greater for the individual starts (32.9% ± 4.1 vs. 56.5% ± 2.8, F_2,8_ = 52.09, *p* = 0.000, η^2^ = 0.93). No differences in angular momentum between start techniques was detected for the upper limbs (F_2,7_ = 3.12, *p* = 0.108, η^2^ = 0.47).

**Table 2:** Total, intrinsic and orbital normalized angular momentum (s^−1^•10^−3^) of the total body and segments (mean ± SE) during individual and relay swimming starts.

		Individual		Relay	*p* value	Effect size
Body	Total	127.8 ± 6.5	<	152.9 ± 4.4	0.004	0.610
	Intrinsic	11.4 ± 1.2	<	16.8 ± 1.2	0.010	0.730
	Orbital	116.4 ± 5.6	<	136.1 ± 4.1	0.012	0.520
Trunk-head	Total	39.1 ± 4.1	<	74.0 ± 3.1	0.001	0.921
	Intrinsic	1.9 ± 1.2		-0.1 ± 1.7	0.110	0.288
	Orbital	37.3 ± 4.3	<	74.1 ± 3.6	0.001	0.936
Lower limbs	Total	70.7 ± 3.0	>	48.1 ± 6.6	0.005	0.608
	Intrinsic	10.1 ± 0.3	<	16.4 ± 0.9	0.001	0.858
	Orbital	60.6 ± 3.1	>	31.6 ± 6.5	0.001	0.740
Upper limbs	Total	17.4 ± 4.9		26.3 ± 6.0	0.285	0.141
	Intrinsic	−0.3 ± 0.2	<	0.6 ± 0.2	0.028	0.471
	Orbital	17.6 ± 5.0		25.7 ± 5.9	0.323	0.122

Note: < and > denote significant differences between individual and relay swimming starts (p < 0.05)

**Figure 4:**
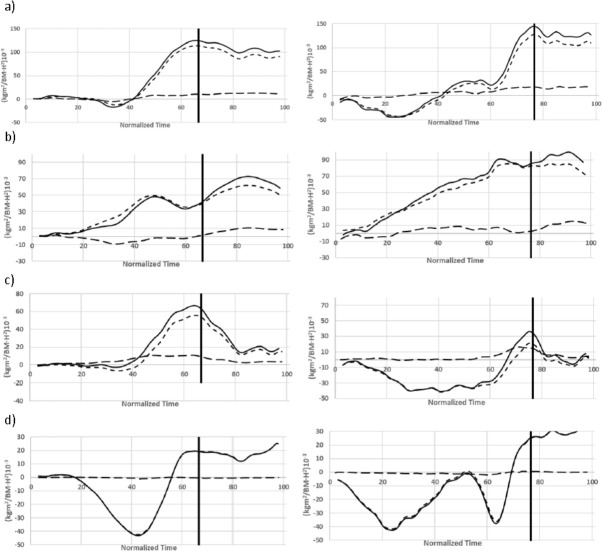
Total, intrinsic and orbital normalized angular momentum of a) the total body, b) the trunk-head, c) the lower limbs, and d) the upper limbs during individual (left) and relay (right) swimming start techniques.

Regardless of the type of start, a greater proportion of the total angular momentum corresponded with the orbital component (Figure 4). At the take-off, the intrinsic component contributed with 16.8 ± 1.2 x 10^−3^∙s^−1^ or 11.4 ± 1.2 x 10^−3^∙s^−1^, while the orbital component reached 136.1 ± 4.1 x 10^−3^∙s^−1^ or 116.4 ± 5.6 x 10^−3^∙s^−1^, respectively, for the relay or individual kick starts (Table 2). In terms of body segments, a greater intrinsic contribution of lower limbs (29.5% ± 5.1) was detected in the relay start compared to both the trunk-head and upper limbs (under 4%), whereas in the individual kick start, the trunk-head (13.2% ± 5.7) and lower limbs (14.6% ± 1.6) presented a higher contribution than the upper limbs (−0.9% ± 1.2).

## Discussion

The present study aimed to improve the understanding of the rotational component in competitive swimming starts by investigating the intrinsic and orbital angular momentum of the body segments in the block and aerial phases. The results suggest that the orbital angular momentum provides the largest rotational component in swimming starts and that the total angular momentum generated during the block phase of relay step starts is greater than that of individual kick starts. However, the swimmer's posture at the water entry for the individual kick start appears to be superior to that of the relay step start due to a more favourable position at the take-off.

The national level swimmers in the present study demonstrated a greater lower limb angular position to the vertical at the water entry during individual vs. relay step starts. These data are consistent with a higher angle of lower limbs to the trunk at the water entry during individual kick starts (Taladriz et al., 2016) and may partially explain the lower performance in the underwater sections of relay step techniques compared to individual kick starts (Qiu et al., 2021b, 2024), despite similar linear kinematics of the centre of mass. In fact, a higher position of the lower limbs at the water entry would reduce the hydrodynamic forces that slow down swimmers (Houel et al., 2013). At the take-off instant, swimmers in both the relay and individual start techniques positioned their trunk segments very close to the horizontal, as reported by Shepherd et al. (2023), although the front lower limb showed a slightly lower position in relation to the swimmer's centre of mass in the individual kick start. For the rear lower limb, the use of the back plate allowed a higher take-off position for the individual compared to the relay start. Finally, the position of the upper limbs showed different models as, according to Sheperd et al. (2023), swimmers presented “arms extended towards the water” in the individual kick start, but “arms extended forward” in the relay step start.

During flight, swimmers in the present study showed greater rotation of the front lower limb during individual kick starts, but greater rotation of the rear lower limb and the trunk during relay step starts (Table 1). This was due to positive angular momentum generated by swimmers at the take-off, which rotated the body during the flight phase to achieve a more vertical body position at the water entry, regardless of the start technique. Overall, the amount of total body angular momentum at the take-off was 19.6% greater for relay step starts than for individual kick starts (Table 2), and this represents an important finding of the present research. In fact, previous studies had not reported differences in total body angular momentum between grab (parallel feet) and kick (separated feet) individual starts (Taladriz et al., 2016) or between different step and non-step relay start techniques (McLean et al., 2000). The present results show the theoretical mechanical advantage of the relay over the individual start techniques, not only for linear but also for angular kinematics. If properly executed, the block preparatory movements should provide relay swimmers with additional horizontal momentum compared to the individual start (Takeda et al., 2010), but also additional angular momentum to rotate body segments around the centre of mass during flight.

On the block phase, swimmers initiated the relay step starts by producing counterclockwise rotations of their segments (i.e., negative angular momentum) until 40% of the start time (Figure 3), which then translated into a rapid increase in positive angular momentum from 60% of the start time until the start. The negative angular momentum in the first stages of the preparatory block movements could be similar to the countermovement mechanisms in vertical jumps, where athletes use the elastic energy stored in the muscle groups to increase the application of the vertical impulse (Wilson and Flanagan, 2008). This rapid increase in angular momentum has previously been observed in parallel feet grab starts (Taladriz et al., 2016) and could be related to the extension of the lower limbs before jumping off the block and to the forward movement of the upper limbs after the countermovement arm swing. On the other hand, the total body angular momentum in the individual kick start increased steadily from 40% of the start time, aided by the take-off of the rear lower limb from the back plate (Taladriz et al., 2016).

The contribution of body segments to the total angular momentum depended on the type of starting technique. The trunk-head segment contributed more to the total angular momentum in the relay step start, but in the individual kick start, the lower limbs made the largest contribution (both over 50% of the total body angular momentum). This different rotational mechanism has been reported when comparing the individual kick start with the traditional grab start (Taladriz et al., 2016) and could be explained by the key role of the back plate and different foot positioning on the block. The support of the rear lower limb on the back plate in the individual kick start probably represented a clear advantage for the lower limbs in terms of the leverage arm for the rotational forces over the centre of mass (Takeda et al., 2017). However, in the relay step start where the feet are in a parallel position at the take-off, the lower limbs cannot generate this amount of rotational force and must therefore be assisted by the trunk head segment. This probably explains the tendency for 1) a higher rotation of the trunk during the flight in the relay step start despite a similar entry angle than in individual kick starts, and 2) a higher angular momentum of the lower limbs at the take-off in the individual kick start that results in a greater clockwise angular displacement of the front lower limb during flight and a more vertical position of lower limbs at the water entry.

However, a full understanding of the rotational components of competitive swim starts requires the investigation of both the intrinsic and orbital components of angular momentum (Landau and Lifshitz, 1974). Our results suggest a small role for the intrinsic rotation of body segments during swim starts compared to the orbital component over the centre of mass. In fact, the contribution of the intrinsic momentum to the total angular momentum was lower to 30% in all the segments of the two analysed starts. This could be interpreted considering the small magnitude of the angular displacement of swimmers during the flight phase compared to other sports disciplines such as somersault diving (Miller et al., 1985). According to these results, the angular momentum in swimming starts would be mainly explained by the linear velocity achieved by the body segments during the block time and their lever arm over the centre of mass. Therefore, high levels of horizontal momentum on the starting block, which would accelerate the swimmer's body forward, would also play an important role in the rotational properties of swimming starts. It should be noted, however, that relay step starts showed greater intrinsic rotation of body segments than individual kick starts (Table 2). Again, the parallel foot position of the relay step starts when diving off the block could explain this, as it required a greater intrinsic component of angular momentum at the take-off compared to individual kick starts where swimmers adopted a track position on the block.

As practical implications for coaches and swimmers, the present results confirm the key role of the kick plate in the rotational components of competitive swimming starts. Swimmers should aim to maximise the horizontal impulse on the block, which coupled with feet track positioning at the take-off would provide orbital angular momentum around the body centre of mass to ensure a proper body position at the water entry. In this line, the execution of relay step techniques where swimmers adopt track feet positioning at the take-off should be explored to assist in the rotation of the body segments during the flight phase. Considering parallel feet start techniques (such as the relay step start), swimmers should maximize the rotational forces during preparatory movements on the block and should take care of the body posture at the take-off and entry instants to minimize the drag forces at the water entry. Overall, findings in the present manuscript should be interpreted taking into account some limitations such as a small sample size (although of a high-performance level) and also the fact that components of angular momentum are influenced by the swimmer’s movements during the flight (and not only by the forces applied on the starting block). Future studies should aim to examine the effectiveness of the angular component of individual and relay starts as a function of the swimmers’ velocity after entering the water.

## Conclusions

The total body angular momentum achieved by competitive swimmers in the present study was greater in the relay step than in the individual kick starts, and it presented a different contribution of body segments depending on the starting technique. In fact, the trunk-head segment provided a greater contribution to body rotation in the relay step start (parallel feet take-off position) compared to a greater contribution from the lower limbs (track feet take-off position) in the individual kick start. This probably helped swimmers perform the individual kick start and achieve a better body posture at the water entry (higher lower limb angular position), despite a lower body rotation during the flight phase, and it highlighted the key role of the starting block back plate. Overall, the rotational component of swimming start techniques was based on the orbital rotation of body segments over the centre of mass, linked to their linear momentum, rather than their intrinsic rotation. This would highlight the importance of the horizontal impulse from the block, not only for the linear kinematics of the centre of mass, but also for the rotational component of swimming starts.

## Figures and Tables

**Figure 1:**
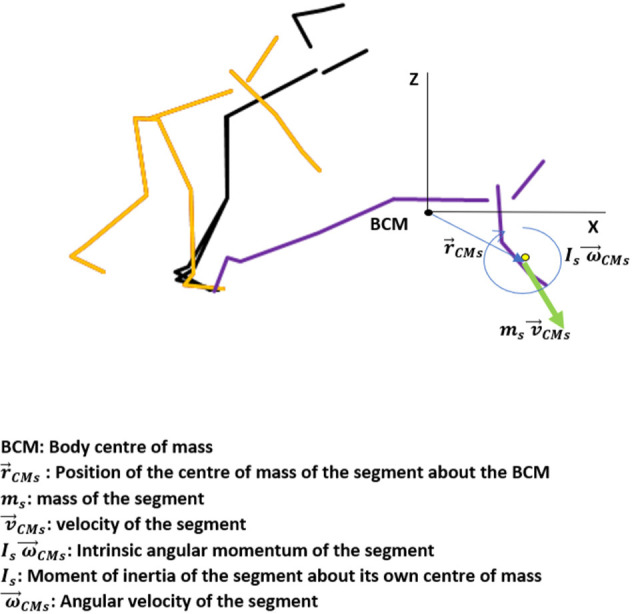
Representation of vectors involved in the angular momentum generated by the swimmer forearm segment on a swimming start take-off.

## Annex 1: An illustrated example of the role of the intrinsic and orbital components of angular momentum in a swimming start

There are previous works in the literature analysing the angular momentum of the body when performing sports techniques (Bahamonde, 2000; Martin et al., 2013; Miller et al., 1985). Most of them have calculated the angular momentum taking into account the intrinsic and orbital components, but to our knowledge, no previous analysis has been carried out on each of these components separately when studying swimming starts. The contribution of the intrinsic rotation or the orbital rotation can play different roles depending on the sport technique. In the supplementary figure, the angular velocity and linear momentum of the swimmer's forearm during a swimming start are shown at the instant of the take-off. Using the original data of the present manuscript, a case was found where the forearm with an angular velocity of 18.5 rad/s (clockwise rotation) produced intrinsic angular momentum of 0.07 kg•m^2^/s (clockwise rotation). Meanwhile, the linear momentum was x = 3.5 kg•m/s, z = −3.9 kg•m/s, producing orbital angular momentum of 1.2 kg•m^2^/s, also clockwise. This example illustrates how the linear velocity of the segment and its position around the centre of body mass can produce greater angular momentum in one direction or another. Consequently, from a practical point of view, the angular components of the swimming start techniques should be analysed taking into account both the local rotation of the segments and their rotation around the centre of mass of the subject.
